# Oral Manifestations and Dental Management of Epidermolysis Bullosa Simplex

**DOI:** 10.5005/jp-journals-10005-1321

**Published:** 2015-09-11

**Authors:** Lisa Scheidt, Mariane Emi Sanabe, Michele Baffi Diniz

**Affiliations:** PhD, Institute of Dentistry, Pediatric Dentistry Postgraduate Program, Cruzeiro do Sul University—UNICSUL, Rua Galvao Bueno, Liberdade, Sao Paulo, Brazil; Assistant Professor, Department of Pediatric Dentistry, Camilo Castelo Branco University—UNICASTELO, Rua Carolina Fonseca, Itaquera Sao Paulo, Brazil; Assistant Professor, Institute of Dentistry, Pediatric Dentistry Postgraduate Program, Cruzeiro do Sul University—UNICSUL, Rua Galvao Bueno, Liberdade, Sao Paulo, Brazil

**Keywords:** Epidermolysis bullosa simplex, Oral manifestations, Alternative therapies.

## Abstract

Epidermolysis bullosa (EB) is a group of hereditary chronic disorders, characterized by fragility of the skin and mucous membranes in response to minor mechanical trauma. The objective of this study was to report the case of a young girl diagnosed with epidermolysis bullosa simplex (EBS), transmitted by an autosomal dominant gene. Cutaneous findings included blisters and dystrophy following minimal friction. Recurrent blisters and vesicle formation on the hard palate were the main oral findings. In conclusion, publications concerning the oral and clinical manifestations of EBS are important for providing knowledge and an early multidisciplinary approach that prevents blister formation and improves these patients’ quality of life, with the dentist playing an important role in oral health management.

**How to cite this article:** Scheidt L, Sanabe ME, Diniz MB. Oral Manifestations and Dental Management of Epidermolysis Bullosa Simplex. Int J Clin Pediatr Dent 2015;8(3):239-241.

## INTRODUCTION

Epidermolysis bullosa (EB) is a heterogeneous group of hereditary disorders characterized by extreme fragility of the skin and mucous membranes, which gives rise to the formation of blisters following minor trauma.^1^ This dermatological condition is a severe autoimmune disease.^2,3^

There are four major types of EB that differ phenotypi-cally and genotypically: simplex (EBS), junctional (JEB), dystrophic (DEB) and Kindler’s syndrome.^4^ Transmission electron microscopy (TEM) is considered the ideal method for diagnosing this pathology.^4^

The most prevalent type of EB is the EBS, which mostly involves feet, hands and neck. Histological analysis reveals that its cleavage level is above the basement membrane.^5^ Local pain is the most common symptom and avoiding friction will prevent lesions.^6^ The maintenance of skin integrity is a serious challenge for dental practice.^7^

Therefore, the aim of this study was to report the case of a girl with EBS, describing the clinical features and the precautions that help improve patient’s quality of life, particularly in relation to dental treatment.

## CASE REPORT

A 10-year-old girl began pediatric dental treatment in 2005 and continued to attend monthly appointments. Her mother authorized the use of her case file for the purposes of scientific studies and signed a term of free, informed consent. The EBS case was diagnosed by a pediatrician soon after birth. Scars on the feet and blisters on the hands showed the need for a precise diagnosis. Therefore, TEM confirmed the autosomal dominant gene through paternal inheritance.

The girl’s diet necessarily includes only soft foods. Oral hygiene has always been performed carefully with an extra soft rubber made toothbrush and fluoride dentifrice.

**Fig. 1:**
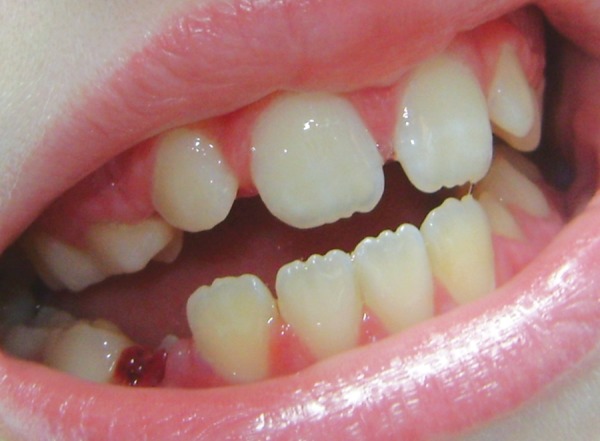
Mixed dentition with no caries and good oral hygiene

**Fig. 2:**
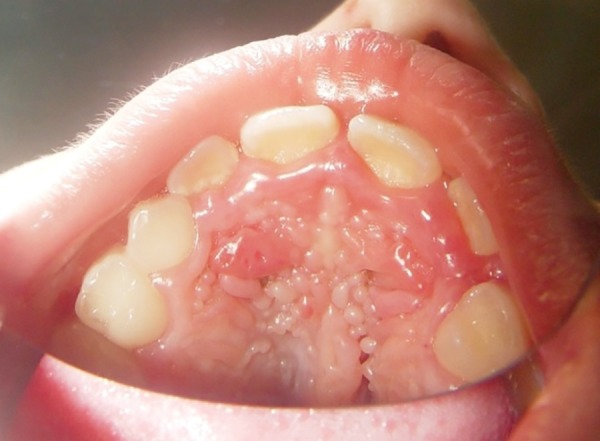
Hard palate and numerous vesicles

**Fig. 3:**
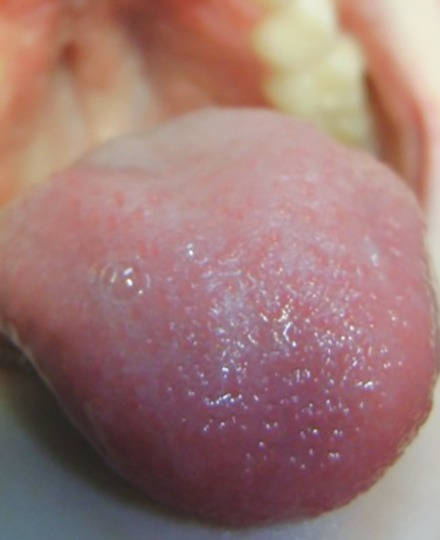
Healthy tongue

Intraoral examination showed mixed dentition (Fig. 1) and the hard palate showed numerous vesicles (Fig. 2), but the tongue presented normal characteristics (Fig. 3). Radiographs were not requested because these lesions affect the skin. The oral manifestations are the same since she began dental treatment.

**Fig. 4:**
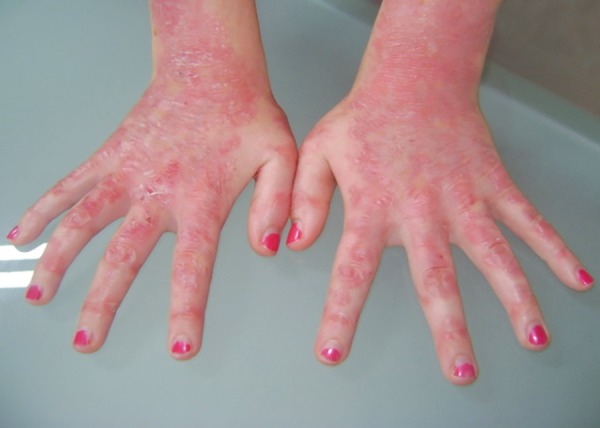
Epidermolysis bullosa simplex manifestations on the hand and fingers

**Fig. 5:**
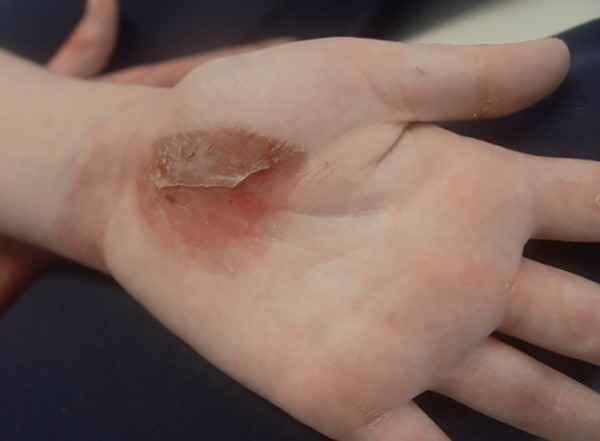
Blister on the palm of the right hand

Her hands were dystrophic (Fig. 4) and usually protected by gloves to avoid any impact. Her right hand showed a blister that she had just perforated (Fig. 5).

The purpose of the dental appointments is to control and prevent caries. The use of an aloe vera tooth gel (bright sparkling, forever living products, Scottsdale, Arizona, USA) at home was suggested to soothe the burning feeling affecting the gums. A mouthwash was also prescribed (Biotene, GlaxoSmithkline, USA) to fortify bioactive enzymes and help the salivary immune system protect the mucosal surfaces.^8^

A diagnosis of EB required monthly dental appointments to maintain a high standard of personal oral hygiene. The recurrent blister lesions continue to develop mostly on the hard palate, but she never had any systemic complications related to EBS.

## DISCUSSION

Epidermolysis bullosa is a challenge to health professionals because there is no definitive cure. Skin care attempts to minimize the severity of blister lesions due to the pain, risk of infection and dissatisfaction with appearance.^2^ Epi-dermolysis bullosa is a prime example of a dermatologi-cal condition that has a profound psychological impact across all aspects of health.^9^ Depression and shame are very common as a result of the appearance.^10^ The patient described in this study is shy.

All major types of EB are characterized by blisters following mild mechanical trauma. Many patients with EB can present systemic complications, such as ocular, genital and oropharyngeal infections, involving difficulty in swallowing.^12^ The patient described in this study was diagnosed early and has not developed any complications or disturbances in swallowing which is in agreement with Fortuna et al.^11^

Epidermolysis bullosa patients require special precautions during dental treatment because of the greater probability of lesioning the soft tissue when handling cutting instruments close to the skin and oral mucosa.^5^ Cariogenic food, limited mouth opening caused by wounds and poor oral hygiene caused by pain are predisposing factors to dental caries.^12^ In this case, minimal intervention has so far preserved the oral cavity and monthly topic fluoride application helped to control dental caries. The patient maintains continuous contact with the health team to avoid complex treatments.

Numerous alternative therapies are used as first aid treatment for blisters. The application of aloe vera gel (bright sparkling, forever living products, Scottsdale, Arizona, USA) diminish the subdermal temperature, providing a refreshed sensation, reducing the healing period and promoting antimicrobial activity.^13^ The decrease in blister formation due to oral moisturizing and saliva stimulation is the reason Biotene mouthwash (GlaxoSmithkline, USA) was prescribed. This product possesses buffering capacity, an immunological effect, antimicrobial activity and a self-cleaning effect.^8^

Epidermolysis bullosa treatment is generally focused on support. Perforating the blisters contributes to accelerating the healing process and prevents continued lateral spread of the blisters. Currently, researchers are focusing their attention on gene and cell therapy, recombinant protein infusions, intradermal injections of allogenic fibroblasts and stem cell transplantation. Other developing therapies are directed toward the enhancement of wound healing and better quality of life for EB patients.^14^

A multidisciplinary approach involving the following health professionals is essential: nutritionist, pediatrician, dermatologist, plastic surgeon, hematologist, gastroentero-logist, ophthalmologist, cardiologist, pediatric dentist, nurse and occupational therapist.

The girl comes to the dental office every month to maintain her oral health. She attends dermatological reevaluations sporadically and, once a year, returns to her pediatrician for control exams. This girl has gotten used to soft food and to avoiding certain physical activities that can hurt her. She believes she has good quality of life, but is always careful not to cause the formation of more blisters.

## CONCLUSION

This case emphasizes that patients with EBS need special precautions during dental treatment because of the greater probability of blister formation. Moreover, those patients require an early multidisciplinary approach to improve their quality of life, with the dentist playing an important role in oral health management.
